# Tissue communication in regenerative inflammatory signaling: lessons from the fly gut

**DOI:** 10.3389/fcimb.2014.00049

**Published:** 2014-04-24

**Authors:** Kristina Kux, Chrysoula Pitsouli

**Affiliations:** Department of Biological Sciences, University of CyprusNicosia, Cyprus

**Keywords:** *Drosophila*, homeostasis, intestine, stem cells, signaling pathways, regenerative inflammatory signaling, tissue communication

## Abstract

The intestine, as a barrier epithelium, serves in the first line of defense against invading pathogens and damaging agents that enter the body via food ingestion. Maintenance of intestinal homeostasis is therefore key to organismal health. To maintain homeostasis, intestinal stem cells (ISCs) continuously replace lost or damaged intestinal epithelial cells in organisms ranging from *Drosophila* to humans. Interestingly, intestinal damage upon ingestion of chemicals or pathogenic bacteria leads to an inflammatory response in the *Drosophila* intestine, which promotes regeneration and predisposes to tumorigenesis. This regenerative inflammatory signaling culminates in proliferation and differentiation of ISCs that replenish the damaged intestinal cells and is regulated by the interplay of conserved cell-cell communication pathways, such as the JNK, JAK/STAT, Wnt/Wingless, Notch, InR, PVR, EGFR, and Hippo. These pathways are induced by signals emanating not only from the damaged intestinal epithelial cells, but also from neighboring tissues associated with the intestinal epithelium, such as the muscles and the trachea, or distant tissues, such as the wounded epidermis and the brain. Here we review tissue communication during homeostasis and regenerative inflammatory signaling in *Drosophila* focusing on the signals that emanate from non-intestinal epithelial tissues to ensure intestinal integrity.

## The *Drosophila* intestine

Due to its functional, structural and cellular similarity to the human intestine, the *Drosophila* intestine has evolved to an excellent model for studying signaling events that control intestinal homeostasis, which, when deregulated, can cause disease (Pitsouli et al., 2009; Apidianakis and Rahme, 2011; Jiang and Edgar, 2011; Jiang et al., 2011).

**Figure 1:**
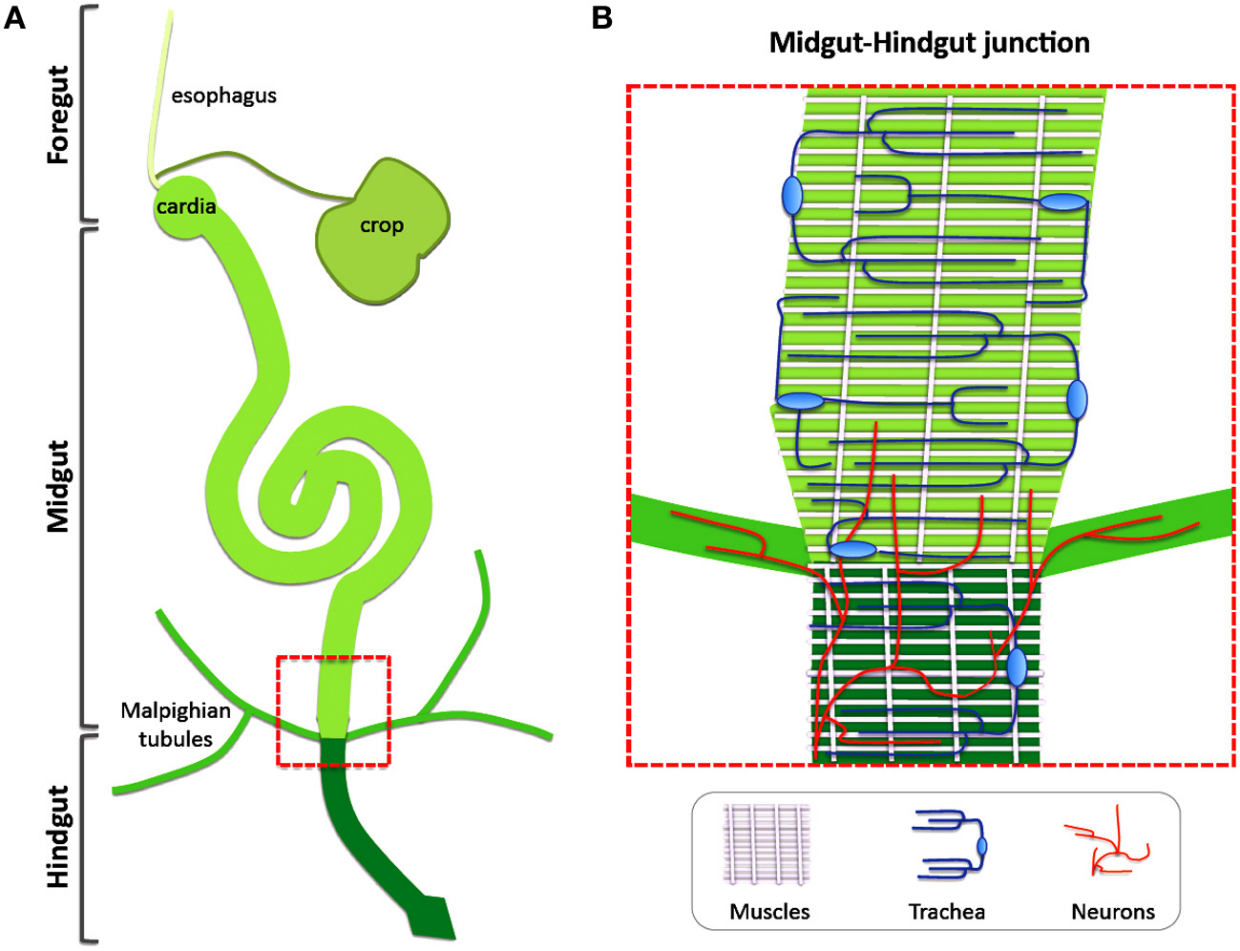
**Non-intestinal epithelial tissues are closely connected with the *Drosophila* gut. (A)** The intestinal tract of the *Drosophila* adult is separated in three main domains: the foregut, the midgut and the hindgut. **(B)** Visceral muscles, intestinal trachea and neurons are integral parts of the intestinal tract. A zoom-up of the boxed area in **(A)** Corresponding to the midgut-hindgut junction is shown to demonstrate the different tissues. Circular and longitudinal visceral muscles ensheath the intestine, tracheal cells generate a vast gas-transporting tubular network around the muscles and neuronal innervations are present at the hindgut-midgut boundary and the malpighian tubules to regulate intestinal physiology.

The adult *Drosophila* intestinal tract is anatomically and functionally separated in three main domains. The foregut, which comprises the esophagus, the crop and the cardia, is followed by the equivalent of the human small intestine, the midgut, and the equivalent of the colon, the hindgut (Demerec, 1950). The intestinal mono-layered tube is ensheathed along its length by circular and longitudinal visceral muscles (VMs) that ensure mixing and grinding, and forward-pushing of the food, respectively (Bayliss and Starling, 1899). The epithelium is covered toward the lumen by the peritrophic membrane (PM), which functions as a structural barrier and contains chitin and glycoproteins (Kuraishi et al., 2011). Between the VM and the intestinal epithelium lies the extracellular matrix-rich basement membrane (BM) (Ohlstein and Spradling, 2006). An extensively ramified network of intestinal trachea responsible for oxygen transport is closely associated with the VMs and reaches the epithelium (Li et al., 2013b). Furthermore, neuronal innervations attach to the esophagus and the cardia, as well as the midgut-hindgut boundary and the rectum, whereas most of the midgut is devoid of innervations (Cognigni et al., 2011) (Figure 1).

The *Drosophila* midgut has recently emerged as a favorite model of intestinal homeostasis. The midgut cells align basally on the BM and are apically separated from the intestinal content by the PM. Four different cell types constitute the midgut epithelium: the differentiated enterocytes (ECs) and enteroendocrine cells (EEs), with absorptive and secretory properties, respectively, the transient enteroblasts (EBs), and the self-renewing intestinal stem cells (ISCs). The ISCs are evenly distributed in the epithelium, reside basally close to the BM and replenish lost cells continuously (Micchelli and Perrimon, 2006; Ohlstein and Spradling, 2006).

## Homeostasis and regeneration in the *Drosophila* midgut

The *Drosophila* midgut is continuously damaged during feeding, as well as by chemicals and pathogens, and needs to be constantly renewed. Homeostatic renewal is ensured via ISC division and differentiation. The ISC division is usually asymmetric and produces two types of daughters: one ISC and one progenitor cell, the EB. The EB does not divide further, but differentiates directly into either an EC or an EE (Micchelli and Perrimon, 2006; Ohlstein and Spradling, 2006, 2007; Jiang and Edgar, 2011).

Intestinal homeostasis is coordinated by the combined action of conserved signaling pathways. In addition to Notch that controls ISC commitment and differentiation depending on its levels (Micchelli and Perrimon, 2006; Ohlstein and Spradling, 2007; Perdigoto et al., 2011), the Wnt/Wg pathway is an important regulator of ISC maintenance and proliferation (Lin et al., 2008; Lee et al., 2009), the Epidermal Growth Factor Receptor (EGFR) and the Target-of-rapamycin (Tor) pathways regulate basal levels of ISC proliferation, the latter in response to nutrition (Amcheslavsky et al., 2011; Biteau and Jasper, 2011; Jiang et al., 2011; Xu et al., 2011) and the Platelet Derived Growth Factor Receptor (PDGFR)/Vascular Endothelial Growth Factor Receptor (VEGFR) pathway, known as PVR in flies, acts in an autocrine manner to control ISC differentiation (Bond and Foley, 2012).

Strikingly, the *Drosophila* midgut exhibits the remarkable ability to regenerate after damage. Ingestion of chemicals, like bleomycin or paraquat, and enteric infection with *Pseudomonas species or Erwinia carotovora carotovora 15* (*Ecc15*) activate a process of regenerative inflammatory signaling, whereby damaged cells produce inflammatory signals that trigger regenerative pathways to replace lost cells and maintain tissue integrity (Panayidou and Apidianakis, 2013). EC damage results in JNK signaling activation, release of IL6-related cytokines (called Unpaired1–3 in flies), induction of EGFs in the intestinal epithelium, as well as the VM, and secretion of Wg from EBs (Biteau et al., 2008; Apidianakis et al., 2009; Buchon et al., 2009a,b; Jiang and Edgar, 2009; Biteau and Jasper, 2011; Jiang et al., 2011). These in turn activate the JAK/STAT, EGFR/Ras/MAPK, and Wg/Wnt cascades in the ISCs to promote proliferation (Jiang and Edgar, 2009; Jiang et al., 2011; Cordero et al., 2012a,b). The EGFR/Ras/MAPK pathway plays a key role in the proliferative response and it is required for both JNK and JAK/STAT-induced ISC proliferation (Buchon et al., 2010; Jiang et al., 2011). In addition, the Hippo pathway acts as a stress sensor in the intestine and responds to changes in epithelial integrity (Karpowicz et al., 2010; Ren et al., 2010; Shaw et al., 2010; Staley and Irvine, 2012). The PVR pathway mediates the response to oxidative stress and aging (Choi et al., 2008) and the injury-induced BMP/Dpp pathway negatively regulates ISC proliferation during the reversion of regeneration-to-maintenance (Guo et al., 2013).

**Figure 2:**
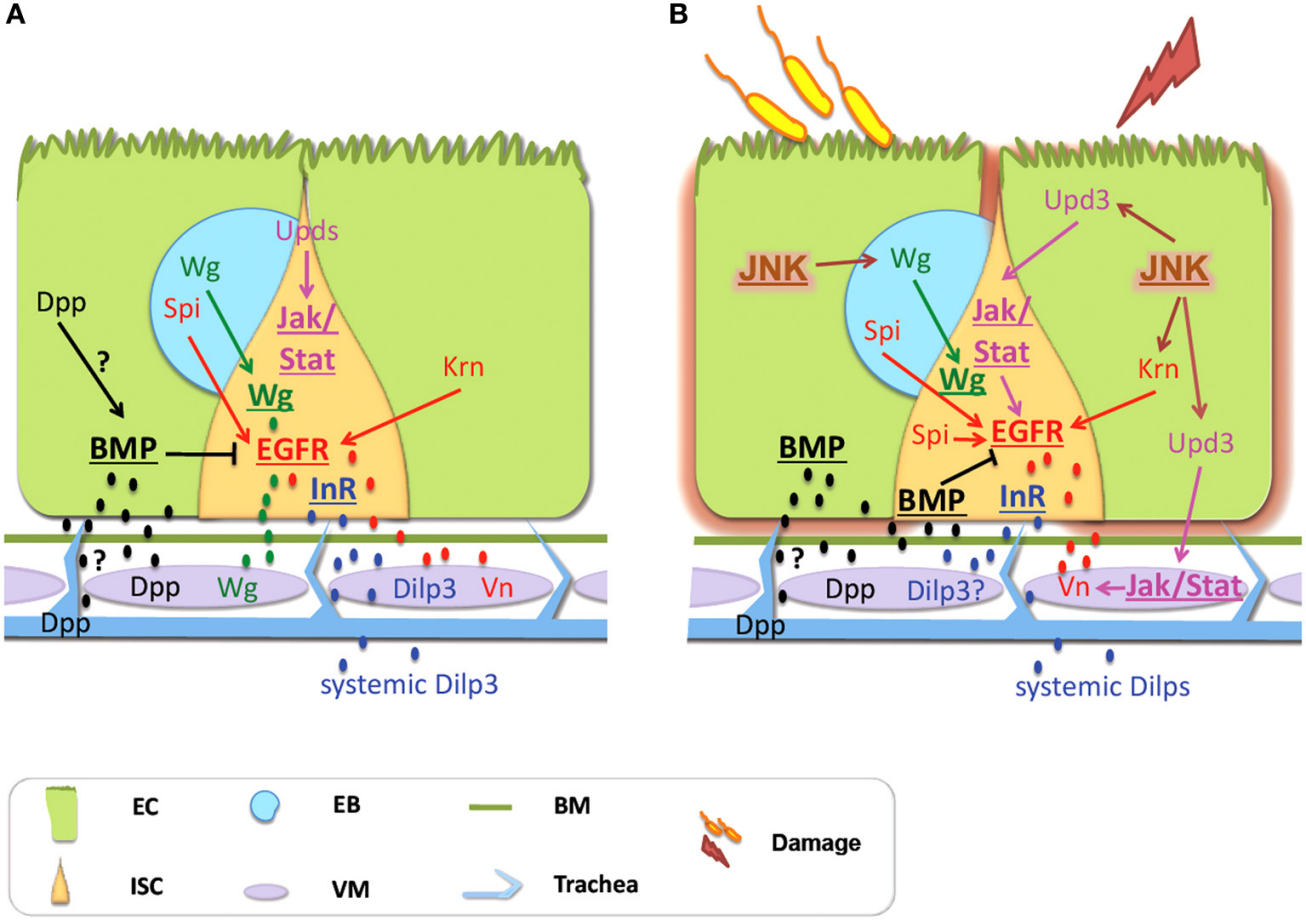
**Signals derived from non-intestinal epithelial tissues control intestinal homeostasis in *Drosophila*. (A)** Physiological Homeostasis: Basal proliferation is controlled via the EGFR and the Wg pathways, whereas JAK/STAT controls differentiation. Vn secreted by the VM, as well as Krn and Spi coming from the EC and the EB, respectively, activate the EGFR pathway. Wg coming from the VM and the EB activates the Wg pathway. Dilp3 secreted from the VM and systemic Dilps activate InR signaling in response to nutrition. Dpp secreted by the VM and possibly by the ECs and the trachea activates the BMP pathway, which inhibits EGFR. Differentiation is regulated by JAK/STAT with the Upd cytokines coming mainly from the ECs. **(B)** Regenerative inflammatory signaling: Enteric infection and ingestion of chemicals induce intestinal damage that promotes regeneration via compensatory ISC proliferation. The EGFR, the Wg and the JAK/STAT pathways control ISC proliferation. JNK signaling is a stress sensor induced in the ECs. It activates EGFR signaling in ISCs and induces Wg in the EBs that activates Wg signaling in the ISCs. The EGFR ligands come from the VM (secreted Vn), the ECs (Krn) and the EBs (Spi). The JNK and JAK/STAT induce proliferation by activating EGFR signaling. Upd3 derived from damaged ECs induces the JAK/STAT pathway in the ISCs and the VM. In the VM, Vn is induced by JAK/STAT activity. BMP signaling is required for the shift from regeneration to basal maintenance; it inhibits EGFR signaling. The BMP ligand Dpp comes from the VM. Additionally, Dpp is expressed in the trachea but this source seems dispensable. Although the InR promotes proliferation, the source and identity of its ligands remain unclear. Signaling pathways are shown in bold and underlined. Abbreviations: EC, enterocyte; ISC, intestinal stem cell; EB, enteroblast; Vn, Vein; Spi, Spitz; Krn, Keren; Wg, Wingless.

Interestingly, the source of the regeneration signals is not confined to the intestinal epithelium. Accumulating evidence suggests that neighboring tissues, such as the muscle, the trachea and potentially the neurons communicate with the intestinal epithelial cells, and thus might function as part of the ISC niche (Figure 2). In the following sections, we review the recent literature on the local and systemic signals emanating from non-intestinal epithelial tissues that ensure intestinal homeostasis during basal tissue maintenance and regenerative inflammatory signaling in *Drosophila*.

## The visceral muscle: a source of Wg, EGFs, Upds and insulin-like peptides

### Wnt/Wg signaling

The first report of inter-organ communication between the adult intestinal epithelium and neighboring tissue, which serves as a functional “ISC niche,” came from a study investigating the role of Wnt/Wg signaling in gut homeostasis (Lin et al., 2008). The authors observed *wg* gene expression in the VM and Wg protein accumulation between the VM and the BM suggesting that VM-secreted Wg reaches the ISCs through the BM. Loss of *wg* significantly reduced the ISC number, whereas ISC clones lacking the Wg receptors or core components of the pathway contained fewer ISCs, suggesting that paracrine VM-produced Wg induces the pathway in the ISCs to promote their self-renewal (Lin et al., 2008). Furthermore, careful analysis of *Adenomatous polyposis coli* (*Apc*) mutant clones, which activate Wnt/Wg signaling, has uncovered a proliferative, not a self-renewal, role of Wg in the ISCs (Lee et al., 2009). Nevertheless, the proliferative effect of Wnt/Wg signaling is mild and was later shown that Wnt/Wg, EGFR and JAK/STAT cooperatively regulate homeostatic ISC proliferation and maintenance (Xu et al., 2011).

Interestingly, a recent report showed that Wg coming from the VM and additional Wg from the epithelium act in concert to regulate ISC maintenance and self-renewal in unchallenged flies (Cordero et al., 2012b). Strikingly, intestinal damage triggered by ingestion of Dextrane Sulfate Sodium (DSS) or *Pseudomonas entomophila* caused Wg upregulation exclusively in the EBs and not the VM. Elegant tissue-specific *wg* inactivation experiments (in the VM and the epithelium), instead of the temperature sensitive *wg* mutation that broadly removes *wg* (Lin et al., 2008), showed that EB-secreted Wg signals to neighboring ISCs to activate downstream pathway components and ISC proliferation (Cordero et al., 2012b).

### EGFR/Ras/MAPK and JAK/STAT signaling

The EGFR pathway was initially shown to regulate development of the midgut epithelium by controlling the proliferation of the adult midgut progenitors (AMPs) (Jiang and Edgar, 2009). Several independent studies subsequently established its key role in ISC proliferation during homeostasis and regeneration (Buchon et al., 2010; Biteau and Jasper, 2011; Jiang et al., 2011; Xu et al., 2011). The three EGFs, Vein (Vn), Spitz (Spi) and Keren (Krn) trigger the EGFR pathway activity in the adult intestine. Vn is expressed in the VM (Buchon et al., 2010; Biteau and Jasper, 2011; Jiang et al., 2011; Xu et al., 2011), whereas Spi and Krn are expressed in the midgut epithelium. Overexpression of *vn, spi* or *krn* in the VM, ISCs/EBs or ECs is sufficient to induce ISC proliferation (Buchon et al., 2010; Jiang et al., 2011; Xu et al., 2011). Nevertheless, there are conflicting reports regarding the necessity of each of the three EGFs in ISC proliferation. Although Jiang et al. (2011) report that neither VM-specific *vn* RNAi nor ISC/EB-specific *spi* RNAi produce an effect, other groups report effects on proliferation (Buchon et al., 2010; Biteau and Jasper, 2011; Xu et al., 2011) and long-term ISC maintenance (Xu et al., 2011) in VM-specific *vn* RNAi. Clearly, the EGFR ligands function redundantly in the *Drosophila* intestine: removing them in combinations produces stronger effects and overexpression of one can rescue loss of another, i.e., overexpression of secreted *spi* in the VM can rescue *vn* RNAi (Xu et al., 2011).

The EGFR ligand redundancy is also observed in stressed or damaged intestines. For example, in response to enteric infection with *Ecc15 vn* is strongly induced in the VM and VM-specific *vn* knockdown impairs ISC proliferation (Buchon et al., 2009b, 2010; Zhou et al., 2013), albeit not fully indicating the redundant function of VM *vn* with other EGFs (Zhou et al., 2013). Indeed, impaired proliferation was also observed by loss of *spi* or *krn* in progenitor cells (Buchon et al., 2010). In addition, VM *vn* is necessary for the ISC regenerative response to damage with paraquat or bleomycin (Biteau and Jasper, 2011). Furthermore, *Pseudomonas entomophila* oral infection leads to induction of *vn* in the VM, *spi* in ISCs/EBs and *krn* in ECs, but only the simultaneous knockdown of *krn* with *spi* or with *vn* impairs stress-induced proliferation underscoring redundancy in EGF function (Jiang et al., 2011).

Both the EGFR and the JAK/STAT pathways are activated during regenerative inflammatory signaling and emerging evidence suggests their interplay at the level of ligand induction. Earlier studies agree that JAK/STAT primarily acts autonomously in the ISCs to regulate their proliferation and differentiation in response to damage (Buchon et al., 2009a,b; Cronin et al., 2009; Jiang and Edgar, 2009). Closer examination of the cell-type specific expression and function of the JAK/STAT ligands has shown that the Upds are induced in distinct cell types: *upd1* is expressed in ISCs/EBs (Osman et al., 2012) and possibly the longitudinal VM (Lin et al., 2010) and it is moderately induced upon bacterial ingestion (Jiang and Edgar, 2009; Osman et al., 2012), *upd2* is probably produced by both progenitors and ECs and exhibits an additive effect to *upd3* in epithelial regeneration upon *Ecc15* infection (Osman et al., 2012), and *upd3* is expressed in ECs and it is strongly induced upon infection (Jiang and Edgar, 2009; Osman et al., 2012; Zhou et al., 2013). Intriguingly, recent evidence suggests that JAK/STAT exhibits a non-autonomous effect on ISC proliferation in response to damage via the activation of EGFs in the VM and the EBs. Specifically, the Upd3-activated JAK/STAT signaling induces *vn* in the VM (Buchon et al., 2010; Jiang et al., 2011; Zhou et al., 2013) and *spi* in the EBs (Zhou et al., 2013). The release of Upd3 from damaged ECs and EBs leads to strong induction of STAT92E activity in ISCs/EBs (Buchon et al., 2009b; Jiang and Edgar, 2009; Zhou et al., 2013) and the VM (Buchon et al., 2010; Zhou et al., 2013) and STAT activation in the VM is sufficient to induce *vn* (Jiang et al., 2011), whereas loss of JAK/STAT activity from the VM leads to loss of VM *vn* and reduces ISC proliferation (Buchon et al., 2010). Interestingly, upon infection with *Pseudomonas entomophila* JAK/STAT activity is dispensable for the induction of *vn* in the VM suggesting that additional signals might be involved in its induction (Jiang et al., 2011).

### Insulin signaling

The insulin pathway promotes ISC proliferation and differentiation during feeding, aging and regeneration (Amcheslavsky et al., 2009; Biteau et al., 2011; Choi et al., 2011) in *Drosophila*. Nevertheless, the source of the Insulin Receptor (InR) ligands that control these processes remains largely unknown. Two of the eight *Drosophila* insulin-like peptides, *Dilp3* and *Dilp7*, are expressed in the intestine: *Dilp7* is expressed in intestinal neurons and regulates intestinal physiology (Cognigni et al., 2011), whereas *Dilp3* is expressed in foregut and midgut muscles (Veenstra et al., 2008). Interestingly, VM-derived Dilp3, supplemented by systemic Dilps, acts directly on the ISCs via the *Drosophila* InR to promote their proliferation and regulates adaptive midgut growth during food intake via both asymmetric and symmetric ISC divisions (O'Brien et al., 2011). Although the inactivation of brain neurons producing systemic Dilps partially inhibits DSS- and bleomycin-induced midgut regeneration (Amcheslavsky et al., 2009), it remains to be tested if intestinal Dilps are also involved.

## The intestinal trachea: a source of Dpp?

Oxygenation of the adult *Drosophila* intestine is achieved via a highly ramified tracheal network overlaying the musculature. The importance of the trachea for intestinal development was highlighted in the silkworm, *Manduca sexta*, where the tracheal and intestinal epithelia grow co-ordinately during metamorphosis (Nardi et al., 2011). In *Drosophila*, tracheal cells project fine extensions through the VM of the adult intestine, which closely contact the intestinal epithelium to allow gas exchange (Li et al., 2013b).

A role of BMP/Dpp signaling in *Drosophila* intestinal homeostasis was first described during larval development, when Dpp is required to keep AMPs undifferentiated (Mathur et al., 2010). Recently, the first study investigating the role of BMP/Dpp signaling in *Drosophila* adult intestinal homeostasis (Li et al., 2013b) showed that loss of BMP/Dpp signaling from the ECs results in ISC proliferation mediated via the ectopic activation of EGFs (*spi* in the ISCs, EBs, ECs, and the VM; and *vn* in the VM). Interestingly, expression of the Dpp ligand is found in tracheal cells and trachea-specific *dpp* RNAi knockdown leads to reduced BMP/Dpp activity in the intestinal epithelium concurrent with increased ISC proliferation suggesting that trachea-derived Dpp is necessary for midgut homeostasis by counteracting stress factors and protecting ECs from apoptosis (Li et al., 2013b).

Interestingly, two additional studies investigating the role of Dpp in intestinal maintenance and regeneration arrived to different conclusions. Guo et al. (2013) report regional differences in *dpp* expression: strong *dpp* in the circular VM of the middle midgut, highly variable *dpp* in the circular VM of the anterior and posterior midgut, and *dpp* expression in the intestinal trachea of unchallenged flies, whereas Li et al. (2013a) report regional graded *dpp* expression in ECs of the middle midgut, but not in the VM or the trachea. Nevertheless, both studies agree that paracrine Dpp acts on ISCs of the middle midgut (the source of the ligand may be both the VM and the ECs) and is necessary and sufficient for the differentiation of specialized midgut ECs, the copper cells (Guo et al., 2013; Li et al., 2013a). Furthermore, intestinal inflammation caused by bleomycin or paraquat induces *dpp* strongly along the midgut in the VM and trachea and leads to BMP/Dpp signaling activation in most ECs and ISCs (Guo et al., 2013). Using highly VM-specific drivers to knockdown *dpp*, Guo et al. (2013) observed strong reduction of BMP/Dpp activity in the midgut suggesting that VM-derived *dpp* is required to induce and maintain the BMP/Dpp signaling. Intriguingly, inactivating *dpp* by RNAi in the VM, but not in the trachea, impaired BMP/Dpp activity in ISCs and led to their proliferation, whereas the proliferative effect observed by depleting downstream components of the BMP/Dpp pathway in ECs (Li et al., 2013b) could not be reproduced (Guo et al., 2013). Since Li et al. (2013b) aged the flies significantly to assess the effects of trachea-specific Dpp knockdown, and the aging intestine exhibits increased intestinal regeneration (Biteau et al., 2008), the age of the flies might have contributed to the observed discrepancies. Furthermore, differences in the genetic background, the diet and the intestinal microbiota of the flies maintained in different laboratories could have also contributed.

## Epidermal injury, distant from the intestine, induces intestinal regeneration

Intriguing recent findings by Takeishi et al. (2013) indicate that aseptic trauma of the adult epidermis induces a systemic wound response that causes renewal of the intestinal epithelium necessary for survival. Specifically, wounding induces ROS in the ECs, followed by caspase-dependent EC apoptosis, which leads to *upd3* activation, ISC proliferation and intestinal regeneration. If caspase activity is blocked in the ECs, regeneration is inhibited and the flies succumb to the trauma leading the authors to suggest that EC apoptosis is essential to counteract a lethal factor present in the hemolymph of wounded flies. Although the nature of the lethal factor remains unknown, it seems that the intestinal response to epidermal injury acts in parallel to ROS-mediated neuronal JNK activation that protects the organism from trauma (Nam et al., 2012).

## Conclusions-perspectives

Although the role of the intestinal nervous system in regenerative inflammatory signaling remains unclear, accumulating evidence in *Drosophila* suggests a key function of the intestinal neurons in physiology. Parallel to systemic signals, gut-specific innervations regulate food intake, fluid and ion balance, as well as physiological intestinal responses triggered by diet or internal metabolic changes (Cognigni et al., 2011). Strikingly, nutrient- and oxygen-responsive neurons, through insulin- and VIP-like peptides, regulate the growth and plasticity of the intestinal tracheal system (Linneweber et al., 2014). Therefore, the nervous system, the trachea and the intestine are intimately connected to maintain physiological homeostasis. Since infection and tumorigenesis affect gut physiology and excretion in *Drosophila* (Apidianakis et al., 2009), it will be interesting to test if the intestinal neurons are implicated in regeneration.

An emerging theme in intestinal regeneration of both *Drosophila* and mammals is the interplay of different signaling pathways that coordinate ISC activity during physiological and regenerative homeostasis. Strikingly, regulatory signals exchanged between the epithelium and surrounding tissues control intestinal maintenance. In *Drosophila*, homeostasis, physiology and regenerative inflammatory signaling are regulated by signals secreted from the intestinal VM (Wnt/Wg, IL6/Upds, EGFs, insulin-like peptides, TGF-beta/Dpp), the trachea (TGF-beta/Dpp) and the neurons (insulin-like peptides, neuropeptides). In mammals epithelial-mesenchymal interactions involving Hh, PDGF, and BMP signaling drive the modeling of the epithelium (Crosnier et al., 2006). Paneth cells, which constitute part of the intestinal niche, express essential regulatory signals, like EGF, TGF-a, Wnt3 or Delta-like-4, which directly control ISC proliferation (Sato et al., 2009, 2011), and stromal cells secrete IL6 (Rigby et al., 2007; Grivennikov et al., 2009; Jiang and Edgar, 2012). In addition, the intestinal subepithelial myofibroblasts, which ensheath the intestinal epithelial cells and closely contact the enteric neurons, express IL23, Wnts and VEGF during inflammation (Andoh et al., 2007), the gut immune cells communicate with intestinal neurons during inflammation (Buhner and Schemann, 2012) to often cause changes in their morphology and density that relate to pathophysiology of the disease, i.e., pain (Demir et al., 2013). Finally, the intestinal blood vessels change their morphology in response to inflammatory signals (Cromer et al., 2011). These observations further underscore the signaling homologies between *Drosophila* and mammals in intestinal homeostasis and regenerative inflammation. Clearly, studies in the *Drosophila* intestinal system will broaden our understanding of tissue communication in mammalian homeostasis.

### Conflict of interest statement

The authors declare that the research was conducted in the absence of any commercial or financial relationships that could be construed as a potential conflict of interest.
